# Remote online global health education among U.S. medical students during COVID-19 and beyond

**DOI:** 10.1186/s12909-022-03434-3

**Published:** 2022-05-10

**Authors:** Peter P. Moschovis, Anupama Dinesh, Anna-Sophia Boguraev, Brett D. Nelson

**Affiliations:** 1 Division of Global Health, Department of Pediatrics, Massachusetts General Hospital, Boston, MA USA; 2 Harvard Medical School, Boston, MA USA; 3 Division of Neonatology, Department of Pediatrics, Massachusetts General Hospital, 125 Nashua St Suite 8426, MB Boston, USA

**Keywords:** Global health, Remote learning, Clinical skills, COVID-19 pandemic

## Abstract

**Background:**

Due to the COVID-19 pandemic, the 2021 Harvard Medical School course *Clinical Topics in Global Health* was offered for the first time as a remote class. We sought to understand student and faculty perceptions of the elective and evaluate the perceived effectiveness of teaching global health using an online education platform.

**Methods:**

Following the course, students and faculty were invited to complete a combined total of three online surveys, which consisted of closed- and open-response questions assessing the strengths and challenges of online learning. Data analyses included traditional descriptive statistics, Net Promoter Score calculation, and inductive thematic analysis of qualitative data.

**Results:**

Thirty-two students and eighteen guest faculty (including four international faculty) participated in the course. Highly-rated course components included guest lecturers, practical skill sessions, polls, and case studies. The Net Promoter Score for the course was excellent at 92, and students reported a greater likelihood of pursuing a career in global health because of the course. While students and faculty highlighted limitations of the remote learning platform (lack of community and interactivity), they also commented on increased accessibility and faculty diversity. Most faculty and students recommended a hybrid model for future versions of the course and suggested strategies to address current limitations.

**Conclusions:**

A remote learning platform can effectively deliver global health education, both in the pandemic setting and beyond.

**Supplementary information:**

The online version contains supplementary material available at 10.1186/s12909-022-03434-3.

## Background

Since March 2020, public health guidance has encouraged social distancing measures to prevent the spread of COVID-19 [1]. These measures have significantly impacted education: schools, universities, and professional schools transitioned from a traditional, in-person format to online modes of learning. Medical schools, in particular, have innovatively addressed this challenge by disseminating theoretical knowledge and clinical skills to students via virtual learning platforms [2–5].

The COVID-19 pandemic has also highlighted the critical importance of global health training for future medical professionals. In an increasingly globalized world, medical trainees in high-income countries must be equipped to address health challenges faced around the world [6]. Medical schools and residency programs are beginning to adapt to the increased interest in demand for global health curricula. For example, pediatric residency programs in the U.S. offering international health electives increased from 25 to 52% between 1995 and 2006 [7], and prior to the pandemic, nearly a quarter of all medical students participated in a global health experience during their training [8].

Global health education exposes medical students and trainees to a wide range of international clinical topics, preparing them to respond to global health inequities and issues. Locally, this specialized training has a positive impact on participating medical students and residents and is associated with improved clinical diagnostic skills, decreased reliance on expensive imaging and lab studies, increased interest in future work in primary care and with underserved populations, increased appreciation for public health, and greater cultural sensitivity [9, 10] Globally, it potentially has an even greater impact. Impoverished and minority populations continue to have poorer health outcomes compared to those living in high-income countries. Inequitable health systems have only been worsened by disruptions of health services due to the COVID-19 pandemic [11]. The need for clinicians trained in the diversity of global health issues is substantial and growing [12, 13].

Online and simulation-based learning methods have been previously used to teach global health and clinical skills. For example, the Simulation Use for Global Away Rotations (SUGAR) curriculum uses simulated cases to prepare residents for the emotional challenges of global health rotations. An online training curriculum, Procedural Education for Adaptation to Resource-Limited Settings (PEARLS), was developed soon thereafter to teach trainees how to adapt clinical procedures to resource-limited settings. According to self-reflections on individual learning, this simulation-based training has demonstrated positive learning outcomes and effectively prepared trainees for situations during clinical experiences abroad [14]. Examples of online global health didactics have also been reported globally [2, 15, 16], with successful results. In an online multidisciplinary global health course taught in Mexico, students reported gaining empathy for global health inequities and an understanding of complex global health issues [15]. A virtual telesimulation program at Yale School of Medicine, developed during the COVID-19 pandemic, resulted in positive feedback from students and faculty. Furthermore, there were no statistically significant differences in evaluation results between students who completed the virtual program compared to those who completed in-person training [17, 18]. Online courses during both the COVID-19 pandemic and the 2014-15 Ebola epidemic in West Africa were successful in providing standardized education [2, 17–19].

Substantive research on transferring clinical knowledge and practical skills remotely to medical students and residents is limited. Previous research indicates that online health education has demonstrated no significant difference in student satisfaction compared to in-person learning and even has demonstrated improved performance in student evaluations of knowledge and skills; [3, 20, 21] however, there remain significant gaps in the literature when considering transferring practical clinical skills in global health. A review of online learning methods in health education reported that although e-learning generally facilitates learning through flexibility and adaptability to individual learning styles including in the clinical applications of health education, it may not be a suitable method of learning for disciplines that require practical demonstrations [22]. Although student perceptions of online clinical teaching methods have been comparable to in-person methods, performance has depended on self-directed student engagement with online learning materials [23, 24]. There remain several knowledge gaps relevant to remote global health education: (a) whether clinical global health skills can effectively be taught via an online platform, (b) whether faculty and students would find a remote platform to be an acceptable method of instruction, and (c) whether the advantages of online learning outweigh its disadvantages.

The elective course presented here, *Clinical Topics in Global Health* at Harvard Medical School, aims to provide trainees essential clinical knowledge and skills to work effectively in resource-limited settings. Previous evaluations of the course demonstrated high levels of satisfaction, significantly increased mean knowledge scores at the end of the course, and stronger preparedness of students for global health experiences [25]. The course has traditionally had a significant focus on practical skills sessions, peer-to-peer learning, and interactive group discussions. However, for its offering in February-March 2021, the course was adapted to be taught entirely online, and it was uncertain whether an online approach would have similar effectiveness and acceptability. At the completion of the course, we sought to understand student and faculty perceptions of the global health elective and the perceived effectiveness of remote teaching of essential global health knowledge and skills.

## Methods

### Course

Since its first offering in 2010, *Clinical Topics in Global Health* has consisted of 10 evening sessions, with each twice-weekly session lasting 2.5-3 h [25, 26], for a total of 25–30 teaching hours. The aim of the course has been to introduce students — primarily preclinical and clinical medical students, but also cross-registered students from other Harvard-affiliated graduate schools and clinical residencies — to the evidence-based knowledge and skills they will need to be effective clinicians in resource-limited settings. Course content covers the leading causes of morbidity and mortality in low- and middle-income countries, including, for example, malnutrition, malaria, diarrheal illnesses, perinatal illnesses, tropical diseases, HIV/AIDS, tuberculosis, and chronic non-communicable diseases. The course also includes discussion of health systems, healthcare delivery, humanitarian assistance, global health ethics, and career development in global health. Each session is accompanied by background readings, principally taken from the non-required textbook, *Essential Clinical Global Health*, which had been developed by over 100 faculty and students from around the globe, largely from earlier offerings of the course [27].

Historically, the course was taught in person and included essential didactic lectures, guest speakers, practical skills sessions, tropical microscopy, and case discussions. However, beginning with the course offering in February-March 2021, in the midst of the ongoing COVID-19 epidemic, the course was necessarily changed to a completely synchronous online course using the Zoom (San Jose, CA, USA) video-conferencing platform. The 10 evening sessions of the 2021 course offering are outlined in Table 1. Platform features such as live polling and virtual breakout rooms were utilized when possible. Meanwhile, participatory skills sessions remained a central component of the course, and thus, hands-on learning required students to find common household items for the simulations (Table 2). For example, in place of the resuscitative equipment and newborn mannequins used for basic newborn resuscitation training in previous years of the course, each student was asked to bring an empty shampoo bottle (or similar) to simulate a self-inflating bag-mask device, hand towels for drying the newborn, and something to roughly approximate a newborn mannequin, such as a relevant-sized doll, stuffed animal, or even a rolled towel. For a large majority of the sessions, local and international topic experts were recruited as guest speakers.

**Table 1:** Course outline, February-March 2021. Schedule sequence of some topics was determined by expert availability

Session 1
• Course welcome (introductions, syllabus, textbook, readings, course website, etc.)
• Introduction to global health
• Global health disparities and non-communicable diseases
• Introduction to international child health
Session 2
• Diarrheal illnesses
• Rehydration techniques
• Practical skills session: making oral rehydration solution from household ingredients
Session 3
• Health care delivery
• Improving post-hospital discharge care and survival
• Global neurology
Session 4
• Humanitarian emergencies
• Malnutrition
• Practical skills session: screening for acute childhood malnutrition using a mid-upper arm circumference (MUAC) tape (UNICEF)
Session 5
• Maternal health
• Practical skills session: improvising a condom-based uterine balloon to manage postpartum hemorrhage (Burke)
• Newborn health
• Practical skills session: basic newborn resuscitation
Session 6
• HIV/AIDS
• Tuberculosis
Session 7
• Malaria
• Acute respiratory infections
• Practical skills session: making a water-bottle spacer for an asthma inhaler [28]
Session 8
• Critical care medicine in resource-limited settings
• Global oral health and essential dental procedures for non-dentists (taught by faculty from the Harvard School of Dental Medicine)
Session 9
• Tropical medicine and neglected tropical diseases
• Infections in returning travelers
Session 10
• Training opportunities and careers in global health
• Discussion panel with three global health practitioners discussing and answering students’ questions related to career models, balancing family-work-travel, global health ethics, etc.

**Table 2:** Common household items required for the practical skills sessions

Making oral rehydration solution from household ingredients	• One heaping teaspoon of sugar • One large pinch of salt• Drinking glass of water (approximately 9 oz) • Spoon
Screening for acute childhood malnutrition using a mid-upper arm circumference (MUAC) tape	• Printed copy of mid-upper arm circumference (MUAC) tape from the syllabus
Basic newborn resuscitation	• Something to roughly simulate a newborn mannequin (e.g., newborn doll, stuffed animal, or even a rolled-up towel) • Something to roughly simulate a bag-mask device (e.g., empty 16 oz water bottle, 1-2-liter water bottle, shampoo bottle) • Two baby blankets, handcloths, dishtowels, or t-shirts
Making a water-bottle spacer for an asthma inhaler	• Empty plastic water bottle (e.g., 500 mL bottle that can be cut with a knife) • Utility knife or kitchen knife

### Study design and study participants

The study used a prospective cohort design. Invited study participants included all students and faculty participating in the course.

### Course evaluation

To evaluate the perceived effectiveness of the course and its new video-conference-based approach, we utilized three web-based anonymous survey tools administered at the completion of the course. The first was a traditional course evaluation consisting of 19 closed-response questions (see Supplemental Digital Appendix 1 for survey instrument) administered independently by our institution to all students. This institutional survey instrument was a well-established, previously-validated instrument used by the school for all electives and covered, for example, student satisfaction, course strengths, course weaknesses, and faculty professionalism. The remaining two surveys — one for all the course’s students and one for all the course’s faculty — were our own REDCap-based (Nashville, TN, USA) online questionnaires (see Supplemental Digital Appendices 2 and 3 for survey instruments), which were developed in consultation with two external experts in medical education evaluation and included closed- and open-ended questions related to the course and its remote-learning approach, specifically, the effectiveness of each teaching component and of remote teaching as a whole. Two follow-up email reminders were sent to each non-respondent.

### Data analysis

Quantitative data from the three survey tools were analyzed with Stata 16.1 (College Station, TX, USA). Frequency and proportion were calculated for categorical variables, and mean and standard deviation were calculated for continuous variables. A paired t-test was used to test for significance of mean differences before versus after the course. Statistical significance was set at *p* = 0.05. We also calculated a Net Promoter Score, a well-recognized measure of customer experience and brand loyalty, calculated based on the percentage of ‘promoters’ minus the percentage of ‘detractors.’[29–32]. To determine the Net Promoter Score, we used the question ‘I would recommend this course to my classmates and colleagues,’ assigning ‘strongly agree’ as ‘promoters,’ ‘slightly agree’ as ‘passive,’ and all others (‘strongly disagree’ + ‘slightly disagree’ + ‘neutral’) as ‘detractors.’

Qualitative open-response data were analyzed for themes using an inductive thematic analytic procedure informed by a grounded theoretical approach [33]. This approach uses iterative cycles of data collection and analysis of emergent themes that are grounded in practical experience rather than in preconceived interpretative structures and is well-suited to mixed quantitative/qualitative analyses in medical education research [28, 34]. Independent two-step coding by two researchers (PPM and BDN) was used; all free text was first open coded to identify salient themes, followed by axial coding examining how themes were related [35].

### Ethical review

 This study was reviewed and deemed exempt by the institutional review board of Mass General Brigham (Boston, MA, USA).

## Results

 A total of 32 students enrolled in the course, including participants from Harvard Medical School (*n* = 22, 68.8%), Harvard School of Dental Medicine (n = 4, 12.5%), Harvard T. H. Chan School of Public Health (*n* = 2, 6.3%), Harvard Divinity School (*n* = 1, 3.1%), Harvard College (*n* = 1, 3.1%), and the Harvard-affiliated medical residency programs (*n* = 2, 6.3%). Most students (*n* = 21, 65.4%) were in the first two years of their medical school training program. The majority (*n* = 12, 66.7%) of the 18 guest faculty were based at Harvard University, with two faculty from other U.S. institutions and four from international institutions. Survey response rates were 50.0% (*n* = 16) for the institution-administered student survey, 81.3% (*n* = 26) for the REDCap-based student survey, and 55.6% (*n* = 10) for the REDCap-based faculty survey.

### Quantitative findings

The vast majority of the students rated the course excellent (*n* = 26, 81.3%) or good (*n* = 4, 12.5%), and most (92.3%) indicated they would recommend the course to colleagues. Based on data provided by this question, the Net Promoter Score for this course was 92. Several aspects of the course were well rated by students, including course organization, guest lecturers, and practical skill sessions (Table 3). All students strongly agreed that the guest lecturers were well prepared and knowledgeable, and most students (*n* = 23, 88.5%) strongly agreed that the course was well-organized. Over half (*n* = 14, 53.8%) of students strongly agreed that the course prepared them to work more effectively in resource-limited settings; all remaining students slightly agreed with this statement. Students reported a significant increase in the likelihood of pursuing a career in global health, from a mean of 75.6% prior to the course to a mean of 85.3% after the course (paired t-test *p* < 0.001).

**Table 3:** Student evaluation of course (n = 16–26)

	Strongly disagree	Slightly disagree	Neutral/ ambivalent	Slightly agree	Strongly agree
The course was well-organized	0 (0%)	0 (0%)	0 (0%)	3 (11.5%)	**23 (88.5%)**
This course has prepared me to work more effectively in resource-limited settings	0 (0%)	0 (0%)	0 (0%)	12 (46.2%)	**14 (53.8%)**
The course content is appropriate for my level of training and experience	0 (0%)	0 (0%)	0 (0%)	11 (42.3%)	**15 (57.7%)**
I would recommend this course to my classmates and colleagues	0 (0%)	0 (0%)	0 (0%)	2 (7.7%)	**24 (92.3%)**
The guest lecturers were well prepared and knowledgeable	0 (0%)	0 (0%)	0 (0%)	0 (0%)	**26 (100.0%)**
The practical skill sessions helped clarify the instruction	0 (0%)	1 (3.8%)	4 (15.4%)	**11 (42.3%)**	10 (38.5%)
A video-conferencing platform was an effective way to learn global health knowledge	0 (0%)	2 (7.7%)	2 (7.7%)	8 (30.8%)	**14 (53.8%)**
A video-conferencing platform was an effective way to learn global health practice	2 (7.7%)	**8 (30.8%)**	7 (26.9%)	4 (15.4%)	5 (19.2%)

Among the various remote learning methods utilized in the course, almost all (*n* = 25, 96.2%) students found guest lecturers to be very effective, and most students found the use of polls (*n* = 21, 80.8%) and case studies (*n* = 16, 61.5%) to be very effective (Table 4). Only 26.9% (*n* = 7) of students found breakout rooms to be very effective. Most (*n* = 14, 53.8%) students strongly agreed that a video-conferencing platform was an effective way to learn global health knowledge; by contrast, a minority of students (*n* = 5, 19.2%) strongly agreed that the platform was an effective way to learn global health practice. Most faculty members reported not using several of the features available in the videoconferencing platform, but among those who did, all reported they were very or slightly helpful. While most (*n* = 6, 60.0%) faculty reported that remote teaching is less effective than in-person teaching in delivering global health education, the majority of faculty (*n* = 7, 70.0%) and students (*n* = 21, 80.8%) recommended that future versions of the course use a hybrid model.

**Table 4:** Student (*n* = 26) and faculty (*n* = 10) evaluation of remote course components

**Students**				
	**Not at all effective**	**Slightly Effective**	**Very effective**	
Polls	0 (0%)	5 (19.2%)	**21 (80.8%)**	
Breakout rooms	2 (7.7%)	**17 (65.4%)**	7 (26.9%)	
Practical skill sessions	2 (7.7%)	**12 (46.2%)**	**12 (46.2%)**	
Guest speakers	0 (0%)	1 (3.8%)	**25 (96.2%)**	
Case studies	0 (0%)	10 (38.5%)	**16 (61.5%)**	
**Faculty**				
	**Not at all helpful**	**Slightly helpful**	**Very helpful**	**Did not use**
Polls	0 (0%)	1 (10.0%)	2 (20.0%)	**7 (70.0%)**
Breakout rooms	0 (0%)	0 (0%)	1 (10.0%)	**9 (90.0%)**
Chat	0 (0%)	2 (20.0%)	**5 (50.0%)**	3 (30.0%)
Verbal questions	0 (0%)	2 (20.0%)	**7 (70.0%)**	1 (10.0%)
Ability to deliver lecture while away from campus	0 (0%)	2 (20.0%)	**8 (80.0%)**	0 (0%)

### Qualitative findings

When students were asked to comment on the strengths and weaknesses of the course and of remote learning, the most commonly reported strength themes were the course’s great faculty lecturers (frequency = 25) and the course’s diverse topics and content (frequency = 19) (Table 5). One student wrote, ‘The professors were phenomenal. Also loved all the guest lectures and all the hands-on activities.’ Among other strengths of the course, students reported enjoying the practical skills sessions (frequency = 9), the well-organized nature of the course (frequency = 5), and the welcoming environment provided by course instructors and fellow students (frequency = 4). For example, a student described the course’s strengths as follows: ‘I really appreciated how pragmatic the course is. It gives real-world examples and interventions, which I really appreciated.’

**Table 5:** Themes and codes related to student (*n* = 26) and faculty (*n* = 10) perceptions of the course and remote learning

	Students	Faculty	Total
**Strengths of course**			
Great faculty	25	0	25
Diverse topics / good content	19	0	19
Practical skills	9	0	9
Well-organized	5	0	5
Welcoming environment	4	0	4
Easily understood by wide range of students	3	0	3
Engaging	3	0	3
Polls	1	0	1
Syllabus	1	0	1
Question and answer with speakers	1	0	1
Case studies	1	0	1
Diverse students	1	0	1
**Weaknesses of course**			
Lack of community	3	1	4
Long sessions	3	0	3
Difficulty with receiving learning materials	2	0	2
**Advantages of remote learning**			
Convenient / accessible to location and schedule	19	3	20
Diverse speakers from around the globe	6	1	7
Saves time	4	4	8
Able to review recordings later	2	0	2
**Disadvantages of remote learning**			
Practical skills and hands-on demonstrations difficult	9	1	10
Less interaction between students and speakers	5	4	9
Harder to stay engaged	5	0	5
Faculty can’t read audience / get student feedback	0	4	4
**Recommendations for course**			
More interactive / practical / in-person days	10	1	11
Add didactic content (equity/colonialism, ethics, global surgery, case studies)	7	0	5
Change course schedule	3	0	3
More diverse speakers	2	0	2
Use video-conferencing functions, cameras more	2	0	2
Better tailor course to different learners	1	0	1
Send kit for practical skills	1	0	1

Among perceived weaknesses of the courses, the most common themes included course sessions that were too long (frequency = 3) and a lack of community among students who did not know each other well (frequency = 4). This was particularly apparent during breakout sessions and may have made them less effective.

When specifically asked about the advantages and disadvantages of remote learning, both students and faculty agreed that the virtual format was convenient to their schedules and saved time (frequency = 22). As one student reported in their evaluation, the virtual course ‘allows 3rd/4th-year students much more access, as many are on rotations month-to-month.’ Many students and faculty also agreed that the virtual format allowed for diverse global guest speakers, ‘enriching [their] educational experience.’ Another student noted that ‘[The course has] given students access to guest speakers from all over the world in all kinds of careers (Médecins Sans Frontières in the trenches, academicians doing big research projects, etc.).’

The disadvantage of virtual learning most commonly reported by students was the difficulty in learning practical clinical skills and engaging with hands-on demonstrations through a virtual video platform (frequency = 9). Furthermore, although students greatly appreciated learning from course faculty, both students (frequency = 5) and faculty (frequency = 4) commented on the lack of valuable interaction between students and speakers compared to an in-person format. A guest lecturer reflected on this limited interaction, commenting that it was ‘more difficult to recreate [an] informal discussion feel and allow for breaks or after class informal discussion with speakers and students.’

Finally, the most frequently reported recommendations from students and faculty include adding additional in-person and practical experiences throughout the course. One faculty member suggested ‘a two-part series: first being remote, [and] second being in-person to demonstrate.’

## Discussion

It has been over a year since the COVID-19 pandemic forced a fundamental restructuring of medical education pedagogy. Our findings suggest that global health education, and perhaps medical education in general, should not be seeking a return to an old normal but instead focus on integrating more virtual or remote components into the curriculum to improve access for both students and faculty.

The remote delivery of *Clinical Topics in Global Health* had many clear advantages, including accessibility. The course’s highest-ever enrollment was in 2021, with more than twice the students enrolled compared to previous years, including third and fourth-year students who attended during clinical rotations. Some of this growth may be attributable to increased demand because of increased public attention to global health, although this additional attention requires adapting for students otherwise unable to access such courses.

A second advantage is that the virtual platform allows students to hear from speakers around the world engaged in various facets of global health work. A major critique of Western academic global health is its failure to integrate global voices [36]. There is clear value to using virtual learning to ‘globalize’ global health classes, ensuring that content is taught by individuals with personal and professional ties to the communities about which the students are learning. With 100% of students giving them the maximum possible score on preparation and knowledgeability, the guest lecturers were clearly a major highlight of the class. Such remote platforms could also allow for truly global health classes, where resources at geographically-inaccessible institutions could be made available not just to local students but internationally-based learners seeking to do this kind of work in their own communities.

While virtual delivery had multiple advantages, the transition to an online class was imperfect. The major disadvantages noted by students and faculty were a lack of community, personal interactions, and the difficulty teaching hands-on skills sessions. One student reflected that the course’s only weakness was the lack of community, and a lecturer noted that it was ‘more difficult to recreate informal discussion.’ Based on these comments, it is possible that students may find a remote course to be a less effective approach to choosing a career direction and finding a mentor. Despite these limitations, students had an overwhelmingly positive impression of the course and its educational value. The Net Promoter Score of this course was excellent at 92, exceeding those of popular high-loyalty consumer brands (Starbucks Coffee 77, Apple iPhone 63, Harvard Business School 41) [37]. This positive impression corroborates previous research that student satisfaction and performance in remote health education courses are comparable to in-person learning [3, 20, 21]. Along with the online course’s demonstrated advantages of accessibility and integration of global voices, it may be valuable to incorporate virtual learning methods to clinical global health courses in the future.

There were also multiple easy-to-implement proposals that may mitigate some of these limitations for future course offerings. While video-conferencing platforms offer multiple features to add interactivity to the learning experience, most lectures were delivered using only basic videoconferencing, live chat, and screen-sharing capabilities. Communication and community may be enhanced by encouraging the use of more video-conferencing features such as breakout rooms and polls. Other suggestions included pre-scheduling virtual office hours which would allow students a chance to interact in a more conversational setting with both the instructors and one another, as well as asking students to enable their cameras during class time.

As educational initiatives transitioned to online learning during the COVID-19 pandemic, other evaluations of these educational efforts have also highlighted the positive components of virtual learning and corroborated our findings that both students and faculty appreciate the accessibility and increased opportunity for global collaborations of remote global health education [38, 39]. In particular, in making the field of global health more equitable and engaging with global colleagues, both our course and others in remote global health demonstrated clear advantages of integrating global perspectives and restructuring equitable partnerships in global health by removing the barrier of travel that in-person learning presents [39–41].

The major critique of the class (loss of in-person skills sessions) and the major advantage (access to and diversity of lectures) do not require that one be sacrificed in favor of the other. In fact, the model favored by both students and faculty, a hybrid in-person/virtual approach, balances the accessibility of online lectures with the utility of in-person skills sessions. While most faculty felt that remote teaching is less effective compared to in-person teaching, no faculty recommended resuming only in-person learning. A hybrid model could deliver the majority of lectures online, supplementing with in-person skills exercises. This model could address the major noted limitation of the course regarding difficulty in disseminating practical skills virtually, as well as some of the limitations noted by students and faculty regarding lack of community and informal discussions by providing a space for in-person mentorship and career development. This proposed model would also maintain reported positive components of remote learning, including accessibility of lectures and diversity in global speakers, a particular advantage for global health education.

Our study was limited by sample size, sample demographics, and potentially unique characteristics of the institution that may limit generalizability. While the REDCap student survey response rate was robust (81.3%), the institution-administered student survey and the REDCap-based faculty survey had response rates of 50.0% and 55.6%, respectively. However, these response rates exceeded those of typical online surveys [42–44].

In view of the findings and limitations of this study, future research is needed to determine the optimal balance between online and in-person learning and innovative approaches to hybrid skills training. The virtual offering of this course was consistent with Garrison et al.’s “Community of Inquiry” theoretical framework for online learning in that the course provided an active learning environment designed for high levels of interactions among students and instructors [45]. However, there are several additional theoretical frameworks of learning that may serve to inform future hybrid models of clinical global health education [46]. Future research is also needed to demonstrate the impact of hybrid models in students’ performance in clinical evaluations in global health. Ultimately, the effectiveness of remote or hybrid global health education will be measured by the long-term outcomes of trainees, which will require longitudinal studies.

## Conclusions

With this study, we hoped to evaluate student and faculty perceptions and the perceived effectiveness of teaching global health using a remote online platform. Study participants were predominantly pleased with the course and cited multiple advantages to remote learning. The success of *Clinical Topics in Global Health* suggests that a remote or hybrid model of medical education may be amenable to adaptation for other global health courses. Further research is needed to leverage the strengths while addressing the challenges of remote online global health training.

## Supplementary Information

**Additional file 1.**

**Additional file 2.**

**Additional file 3.**

## Data Availability

All data generated or analysed during this study are included in this published article and its supplementary information files.

## Abbreviations

PEARLS: Procedural Education for Adaptation to Resource-Limited Settings
SUGAR: Simulation Use for Global Away Rotations
