# Supplementary material for: Remote online global health education among U.S. medical students during COVID-19 and beyond
Source: BMC Med Educ. 2022 May 10;22:353. doi: 10.1186/s12909-022-03434-3 (PMC9087168; doi:10.1186/s12909-022-03434-3)
Supplement: Supplementary file 1 — Additional file 1. [file 12909_2022_3434_MOESM1_ESM.pdf]

HARVARD MEDICAL SCHOOL

Preview Evaluation

2020-2021 : Nonclinical Electives : 260-SM520.0 - Clinical Topics in Global Health

Course Evaluation

HMS Nonclinical Elective Course Evaluation Template

Return to Evaluation

Course Information

Date	Course	Location	Weeks	Credits
01/01/2006 - 01/31/2006	XXX-YYY: Department Course	Location	8	8

Evaluation Period: 01/01/2006 - 01/31/2006

This is a standard set of questions used by students to evaluate core courses at Harvard Medical School (HMS). Your responses to these questions are essential to monitor the quality of the educational experience at the Medical School. The identity of all respondents is confidential.

Question numbers in **red*** are required.

SECTION I: Overall Rating

1.* Please rate the course overall.

- ◯ Excellent
- ◯ Good
- ◯ Average
- ◯ Fair
- ◯ Poor

2. Please comment on the **strengths** of the course.

Rich text

3. Please comment on **areas in which this course could be improved.**

Rich text

SECTION II: The questions in this section ask you about your experience of the course design and the types of learning experiences included in the course.

4.* How many **hours each day, on average,** did you spend reading, preparing, or studying for this course?

- ◯ less than 2 hours per day
- ◯ more than 2 but less than 4 hours per day
- ◯ more than 4 but less than 6 hours per day
- ◯ more than 6 but less than 8 hours per day
- ◯ more than 8 hours per day

5.* During this course faculty provided direction and constructive feedback.

- ◯ Never
- ◯ Almost Never
- ◯ Sometimes
- ◯ Fairly Often
- ◯ Very Often
- ◯ Always

6.* I was given the opportunity to ask questions during this course.

- ◯ Never
- ◯ Almost Never
- ◯ Sometimes
- ◯ Fairly Often
- ◯ Very Often
- ◯ Always

7. Please comment on the educational environment created by this course.

Rich text

Please rate the educational elements of the course:

8. Please rate the utility of the prep materials in supporting your learning in the course.

- ◯ Excellent
- ◯ Good
- ◯ Average
- ◯ Fair
- ◯ Poor

9. Please comment about your experience of receiving feedback on your performance from faculty in this course.

Rich text

10. Please comment on which of the educational elements (patient experiences, didactics, small group interactive learning activities, etc.) contributed the most to your learning.

Rich text

11. Please comment on your experience interacting with course content on Canvas, including the organization of materials and ease of locating resources.

Rich text

SECTION III: The questions in this section ask you about your experiences in this course, including interactions with faculty. Please consider all learning experiences in this course when answering these questions (e.g., learning studio sessions, large group sessions in the amphitheater, small group sessions and laboratories, as well as meetings with faculty). Since this is to provide us with an overall sense of the learning environment at HMS, please try to keep in perspective the frequency of positive and negative experiences and interactions with faculty (i.e., if 1 interaction out of 10 was poor, your rating should reflect that frequency).

Thinking about YOUR experiences during the course, please choose one of the categories of frequency provided below. Choose the category that best approximates YOUR perception.

12.* There are disconnects between what I am taught about professional behaviors/attitudes and what I see being demonstrated by faculty.

- ◯ Never
- ◯ Almost Never
- ◯ Sometimes
- ◯ Fairly Often
- ◯ Very Often
- ◯ Always

13.* It felt safe to ask questions during this course.

- ◯ Strongly agree
- ◯ Somewhat agree
- ◯ Neither agree nor disagree
- ◯ Somewhat disagree
- ◯ Strongly disagree

For the remaining questions in this section, please rate how often the following professional behaviors/attitudes were demonstrated by faculty involved in the course.

14.* Showing respectful interaction with students.

- ◯ Never
- ◯ Almost Never
- ◯ Sometimes
- ◯ Fairly Often
- ◯ Very Often
- ◯ Always

15.* Using professional language/avoiding derogatory language.

- ◯ Never
- ◯ Almost Never
- ◯ Sometimes
- ◯ Fairly Often
- ◯ Very Often
- ◯ Always

16.* Resolving conflicts in ways that respect the dignity of all involved.

- ◯ Never
- ◯ Almost Never
- ◯ Sometimes
- ◯ Fairly Often
- ◯ Very Often
- ◯ Always

17.* Being respectful of HMS faculty and HMS staff.

- ◯ Never
- ◯ Almost Never
- ◯ Sometimes
- ◯ Fairly Often
- ◯ Very Often
- ◯ Always

18.* Respecting diversity.

- ◯ Never
- ◯ Almost Never
- ◯ Sometimes
- ◯ Fairly Often
- ◯ Very Often
- ◯ Always

19.* Showing empathy and compassion.

- ◯ Never
- ◯ Almost Never
- ◯ Sometimes
- ◯ Fairly Often
- ◯ Very Often
- ◯ Always

Harvard Medical School and its affiliated institutions take student mistreatment seriously and aspire to a culture of zero tolerance for instances of abuse, mistreatment, and disrespect. By reporting instances of mistreatment, you help the school and hospitals sustain an environment of collegiality and mutual support. Please use the questions below to report any instances of mistreatment that you experienced or witnessed during this course.

As you consider potential instances of student mistreatment, please keep in mind that clinical practice and clinical training involve a high level of accountability to the well-being of patients, and expectations of clinicians and of students are high. Accordingly, part of clinical training may be uncomfortable - e.g., being "put on the spot" on rounds or in the operating room-but does not constitute mistreatment. With this fine distinction in mind (e.g., between appropriate accountability and inappropriate disrespect), please do your best to respond to the following questions.

Please note that reports of mistreatment are embargoed in the system so that course directors are not able to access them. Instead these reports are sent directly to the HMS Dean for Students (who has no role in individual student evaluation) who develops a plan of action to respond to each report individually. This plan may include follow-up with the course director and/or clinical site where the mistreatment occurred, but in all cases the situation will be handled in such a way as to protect the student or students involved.

20.* Have you personally been mistreated during this educational experience?

- ◯ No (if no, you can skip all the additional questions)
- ◯ Once
- ◯ Two or more times

21.* Have you personally witnessed mistreatment of other students during this educational experience?

- ◯ No (if no, you can skip all the additional questions)
- ◯ Once
- ◯ Two or more times

Please use the following descriptions to inform your answers to the next question.

General Mistreatment: Belittled publicly, humiliated, threatened with physical harm or punished physically, required to perform personal services.

Sexual Mistreatment: Asked to exchange sexual favors for grades or other rewards, denied opportunities for training or rewards because of gender, subjected to unwanted sexual advances because of gender, subjected to offensive sexist remarks/names because of gender, received lower evaluations or grades solely because of gender.

Racial/Ethnic Mistreatment: Denied opportunities for training or rewards because of race or ethnicity, subjected to racially or ethnically offensive remarks/names directed to you personally, received lower evaluations or grades solely because of ethnicity.

Sexual Orientation Mistreatment: Denied opportunities for training or rewards because of sexual orientation, subjected to offensive remarks/names directed to you personally, received lower evaluations or grades solely because of sexual orientation.

22. If you experienced mistreatment during this educational experience please indicate of what type; please check all that apply.

- ◯ General Mistreatment
- ◯ Sexual Mistreatment
- ◯ Racial/Ethnic Mistreatment
- ◯ Sexual Orientation Mistreatment

23. If you experienced mistreatment during this educational experience please identify the source of the mistreatment.

- ◯ Preclinical Faculty
- ◯ Clinical Faculty (in classroom)
- ◯ Clinical Faculty (in hospital or clinic)
- ◯ Residents/Interns
- ◯ Nurses
- ◯ Other hospital staff (e.g., pharmacists, custodial staff)
- ◯ Administrators
- ◯ Students
- ◯ Patients

24. Please describe the incident(s).

Rich text

25. Did you formally report your experience(s) of mistreatment to a faculty member, course/clerkship leader, or member of the School administration (e.g. Dean, Society Director, HMS staff person)?

- ◯ No. Please indicate why not below.
- ◯ Yes. Please indicate to whom you reported your experience(s) of mistreatment below.

26. Details of reporting.

Rich text

THANK YOU!

Return to Evaluation

Note: Internet Explorer version 10 and earlier are not compatible with OASIS.

- More information on compatible browsers

Problems? Contact:

Contact the HMS IT Service Desk

- **617.432.2000**
- **itservicedesk@hms.harvard.edu**
- Business hours Monday - Friday, 8am - 5pm
- After Hours urgent issues: call and select option to page on-call technician

POWERED BY OASIS

Copyright © 2008-2021 WARF
