# Supplementary material for: Remote online global health education among U.S. medical students during COVID-19 and beyond
Source: BMC Med Educ. 2022 May 10;22:353. doi: 10.1186/s12909-022-03434-3 (PMC9087168; doi:10.1186/s12909-022-03434-3)
Supplement: Supplementary file 2 — Additional file 2. [file 12909_2022_3434_MOESM2_ESM.pdf]

CTGH Post-Course Survey

Participant ID

____

Please indicate your primary institution:

- ◯ Harvard Medical School
- ◯ Harvard School of Dental Medicine
- ◯ Harvard T.H. Chan School of Public Health
- ◯ Harvard Divinity School
- ◯ Harvard-affiliated resident
- ◯ Harvard-affiliated fellow
- ◯ Other (specify)

Other institution:

____

Please indicate your current year of training within your current training program:

- ◯ 1
- ◯ 2
- ◯ 3
- ◯ 4
- ◯ 5
- ◯ 6 or higher

PRIOR to the course, how likely was global health to be a significant component of your career?

Very unlikely — Uncertain/Neutral — Very likely

(Place a mark on the scale above)

AFTER the course, how likely is global health to be a significant component of your career?

Very unlikely — Uncertain/Neutral — Very likely

(Place a mark on the scale above)

Which of the teaching methods used in the course were effective?

	Not at all effective	Slightly effective	Very effective
Polls	◯	◯	◯
Breakout room	◯	◯	◯
Practical skill sessions	◯	◯	◯
Guest speakers	◯	◯	◯
Case studies	◯	◯	◯

What has this course done especially well?

____

What are some of the weaknesses in this course? Do you have any suggestions for ways we can make the course better or more helpful to you?

____

What do you consider are the advantages and disadvantages of holding this course on a video-conferencing platform?

____

Once in-person learning is possible again, which teaching method would you recommend for this course?

◯ Return to in-person learning on the Harvard Medical School campus ◯ Continue to offer this course on a video-conferencing platform ◯ Use a combination of in-person and video-conferencing sessions

Please indicate your level of agreement with the following statements:

	Strongly disagree	Slightly disagree	Neutral/ ambivalent	Slightly agree	Strongly agree
The course was well organized.	◯	◯	◯	◯	◯
This course has prepared me to work more effectively in resource-limited settings.	◯	◯	◯	◯	◯
The course content is appropriate for my level of training and experience.	◯	◯	◯	◯	◯
I would recommend this course to my classmates and colleagues.	◯	◯	◯	◯	◯
The guest lecturers were well prepared and knowledgeable.	◯	◯	◯	◯	◯
The practical skill sessions helped clarify the instruction.	◯	◯	◯	◯	◯
A video-conferencing platform was an effective way to learn global health knowledge .	◯	◯	◯	◯	◯
A video-conferencing platform was an effective way to learn global health practical skills .	◯	◯	◯	◯	◯

Please indicate whether you agree that the session was useful in preparing you to work effectively in resource-limited settings:

	Strongly disagree	Slightly disagree	Neutral/ ambivalent	Slightly agree	Strongly agree	Did not attend session
Session 1 (Tue, 2/2): Introduction to global health (Nelson)	◯	◯	◯	◯	◯	◯
Session 1 (Tue, 2/2): Introduction to international child health (Nelson)	◯	◯	◯	◯	◯	◯
Session 1 (Tue, 2/2): Global health disparities and non-communicable diseases (Moschovis)	◯	◯	◯	◯	◯	◯

Session 2 (Thur, 2/4): Diarrheal illnesses (Weil)	○	○	○	○	○	○
Session 2 (Thur, 2/4): Rehydration and ORS (Nelson)	○	○	○	○	○	○
Session 2 (Thur, 2/4): Practical skills session - Make your own ORS (Nelson)	○	○	○	○	○	○
Session 3 (Tue, 2/9): Health care delivery (Rhatigan)	○	○	○	○	○	○
Session 3 (Tue, 2/9): Global neurology (Wibecan)	○	○	○	○	○	○
Session 3 (Tue, 2/10): Improving post-discharge care (Wiens)	○	○	○	○	○	○
Session 4 (Tue, 2/16): Malnutrition (Cook)	○	○	○	○	○	○
Session 4 (Tue, 2/16): Humanitarian emergencies (Greenough)	○	○	○	○	○	○
Session 4 (Tue, 2/16): Practical skill session - MUAC (Nelson)	○	○	○	○	○	○
Session 5 (Thur, 2/18): Maternal health (Boatin)	○	○	○	○	○	○
Session 5 (Thur, 2/18): Newborn resuscitation (Nelson)	○	○	○	○	○	○
Session 5 (Thur, 2/18): Practical skill session - Newborn resuscitation (Nelson)	○	○	○	○	○	○
Session 6 (Tue, 2/23): HIV/AIDS (Powis)	○	○	○	○	○	○
Session 6 (Tue, 2/23): Tuberculosis (Carter)	○	○	○	○	○	○
Session 7 (Tue, 3/2): Malaria (Wirth)	○	○	○	○	○	○
Session 7 (Tue, 3/2): Acute respiratory infections in children (Chisti)	○	○	○	○	○	○
Session 7 (Tue, 3/2): Practical skill session - Water bottle spacer (Moschovis)	○	○	○	○	○	○
Session 8 (Thur, 3/4): Critical care medicine in low-resource settings (Riviello)	○	○	○	○	○	○

Session 8 (Thur, 3/4): Clinical oral health for non-dentists (Swann)	○	○	○	○	○	○
Session 9 (Tue, 3/9): Essential tropical medicine/neglected tropical diseases (Ryan)	○	○	○	○	○	○
Session 9 (Tue, 3/9): Travel medicine and immigrant health (Harris)	○	○	○	○	○	○
Session 10 (Thur, 3/11): Trends in global health education and training (Nelson)	○	○	○	○	○	○
Session 10 (Thur, 3/11): Faculty career panel discussion: careers in global health (Various)	○	○	○	○	○	○

Any other comments or suggestions? ____________________________
