# Supplementary material for: Remote online global health education among U.S. medical students during COVID-19 and beyond
Source: BMC Med Educ. 2022 May 10;22:353. doi: 10.1186/s12909-022-03434-3 (PMC9087168; doi:10.1186/s12909-022-03434-3)
Supplement: Supplementary file 3 — Additional file 3. [file 12909_2022_3434_MOESM3_ESM.pdf]

CTGH Faculty Survey

We would appreciate feedback on your experience teaching in Clinical Topics in Global Health this year.

Thank you!

Please indicate your primary institutional affilation:

- ◯ Harvard University (Harvard Medical School/Harvard School of Public Health)
- ◯ Other university/medical center in the US
- ◯ Other university/medical center outside the US
- ◯ Other (specify)

Other institution: ______________________

How helpful were the following teaching methods in delivering your lecture?

	Not at all helpful	Slightly helpful	Very helpful	Did not use (N/A)
Polls	◯	◯	◯	◯
Breakout rooms	◯	◯	◯	◯
Chat Q&A	◯	◯	◯	◯
Verbal questions	◯	◯	◯	◯
Ability to deliver lecture while away from the Harvard campus	◯	◯	◯	◯

In your opinion, what are the advantages of teaching via a remote platform (compared to in-person teaching)? ______________________

In your opinion, what are the disadvantages of teaching via a remote platform (compared to in-person teaching)? ______________________

How effective do you think remote teaching is in delivering global health education, compared to in-person teaching?

◯ Remote teaching is less effective than in-person teaching. ◯ Remote teaching is equally effective as in-person teaching. ◯ Remote teaching is more effective than in-person teaching.

When the course is offered again, would you prefer to teach remotely or in person?

◯ In-person learning on the Harvard Medical School campus ◯ Continue to offer this course on a video-conferencing platform ◯ Use a combination of in-person and video-conferencing sessions

Any additional comments on your experience teaching in this course? ______________________
